# Identification of key pathways and genes in PTEN mutation prostate cancer by bioinformatics analysis

**DOI:** 10.1186/s12881-019-0923-7

**Published:** 2019-12-02

**Authors:** Jian Sun, Shugen Li, Fei Wang, Caibin Fan, Jianqing Wang

**Affiliations:** Department of Urology, The Affiliated Suzhou Hospital of Nanjing Medical University, 26 Daoqian Rd, Suzhou, 215000 Jiangsu China

**Keywords:** Prostate cancer, Bioinformatics analysis, PTEN mutation, TCGA, RNA seq

## Abstract

**Background:**

Prostate cancer (Pca) remains one of the leading adult malignancies. PTEN (Phosphatase and Tensin Homolog) mutant is the top common mutated genes in prostate cancer, which makes it a promising biomarker in future individualized treatment.

**Methods:**

We obtained gene expression data of prostate cancer from TCGA (The Cancer Genome Atlas) database for analysis. We analyzed the DEGs (differentially expressed genes), and used online tools or software to analyze Gene ontology (GO) and the Kyoto Encyclopedia of Genes and Genomes (KEGG), Gene set enrichment analysis (GSEA), Search Tool for the Retrieval of Interacting Genes/Proteins, and Molecular Complex Detection.

**Results:**

Latest TCGA data showed PTEN mutation in about 22% patients. 1736 DEGs in total were identified. Results of gene functional enrichment analyses showed that muscle contraction, negative regulation of growth and multiple metabolic progression were significantly enriched. GNG13, ACTN2, POTEE, ACTA1, MYH6, MYH3, MYH7, MYL1, TNNC1 and TNNC2 were the top ten hub genes. Patients with PTEN mutation showed relatively decreased mRNA expression level of PTEN. Survival analysis indicated the risk of disease recurrence in patients with PTEN mutation.

**Conclusions:**

Our findings suggested that PTEN mutation in prostate cancer may induce changes in a variety of genes and pathways and affect disease progression, suggesting the significance of PTEN mutation in individualized treatment of prostate cancer.

## Background

Prostate cancer is one of the common malignant tumors in urology and is the leading cause of cancer-related mortality in men in developed countries. [1]. Previous studies have focused on signaling pathways, critical oncogenes and related cellular processes that promote disease progression, but the underlying molecular biologic mechanisms are still not clear. To further explore some new mechanisms or treatment targets, various online databases, such as The Cancer Genome Atlas (TCGA) and Gene Expression Omnibus (GEO), offer gene expression data and correlated variable clinical course of mutiple cancer types [2]. However, until now, there have been few researches focused on the individualized treatment based on the datasets above.

Gene mutations have been shown to be critical in disease program. In primary prostate cancer, metastatic prostate cancer, and metastatic, castration-resistant prostate cancer (mCRPC), recurrent somatic mutations, copy number changes, and oncogenic structural DNA rearrangements (chromosomal abnormalities) have been identified [2–5]. The combined results of several studies have confirmed that PTEN is the top common mutated genes in PCa, indicating its potential clinical significance. PTEN is a multi-functional tumor suppressor and, loss of expression of PTEN has been found in about 70% of PCa patients [6, 7]. Functionally, PTEN regulates cell proliferation and survival mainly by regulating PI3K-AKT related signaling pathways [8]. Recent years, latest researches indicated that PTEN could modulate cell growth via AKT-independent mechanisms [9]. Moreover, prostate-specific Pten deletion in mice model results in significantly shortened course of prostatic intraepithelial neoplasia development and promotes metastatic prostate cancer formation [6].

In prostate cancer, PTEN is commonly altered through copy-number loss rather than point mutation. Previous researches have focused on PTEN mRNA expression in prostate cancer initiation and development [10]. Since the PTEN mutation is quite common in prostate cancer patients, we want to know whether the cluster analysis of the tumor molecular spectrum data will define the PTEN mutant tumor as a subgroup. In addition, various critical cell signaling pathways and processes may change in patients with PTEN mutations compared to PTEN wild-type patients [8]. Therefore, different PTEN status may influence disease development, outcome and therapeutic strategies, causing naturally resistant. Further study of the changes in cell processes and pathways in patients with PTEN mutations will contribute to further understanding of the pathogenesis of the disease and provide more novel evidence for the accurate treatment of prostate cancer.

In the present study, we used the data of a RNA sequencing (RNA-Seq) dataset to identify the mechanims underlying PTEN mutation in prostate cancer by bioinformatics analysis, expecting to reveal the potential function of PTEN mutation in predicting prognosis and individualized treatment options.

## Methods

### Data information

We obtained the data and clinical profiles of a prostate cancer RNA-Seq (project: TCGA-PRAD) from TCGA (The Cancer Genome Atlas) database. Other related clinical resourses were downloaded directly from the cBioPortal website (website: http://www.cbioportal.org) [11].

### Analysis of GSEA (gene set enrichment analysis)

To understand the effect of PTEN mutation on various biological function gene sets in prostate cancer patients, GSEA was used to do the enrichment analysis. The number of permutations was set at 5. A *P*-value cutoff of 0.05 with a false discovery rate (FDR q-val) < 0.25 were considered statistically significant [12, 13]. GSEA v3.0 (http://software.broadinstitute.org/gsea/downloads.jsp).

### Identification of differentially expressed genes (DEGs)

We used EdgeR to study gene differential expression of mRNA levels between PTEN mutations and PTEN wild-type prostate cancer patients [14, 15]. Genes with |fold change (FC)| ≥2 and both the *P*-value and FDR <0.05 were identified as DEGs.

### Functional enrichment analysis

Candidate DEGs functions and pathways enrichment were analyzed using R package clusterProfiler. Gene ontology (GO) analysis and pathway analysis were carried out using Gene Ontology website (Available online: http://www.geneontology.org/) and KEGG PATHWAY (Available online: http://www.genome.jp/kegg) with a *P* value cutoff of < 0.05.

### Protein–protein interaction analysis

We used Search Tool for the Retrieval of Interacting Genes (STRING) database to analyze the protein interation network [16]. We submitted the DEGs to the website to evaluate the interactive relationships. Combined score >0.4 were selected as significant. Then, the module screening was completed by Molecular Complex Detection (MCODE) in Cytoscape (scores >3 and nodes >4) [17].

### Statistical analyses

Student’s t-test was used to compare the PTEN mRNA expression level and Gleason score between PTEN mutation and PTEN wild-type prostate cancer tissue. We evaluated the prognosis between different PTEN status groups using the Kaplan–Meier method with log-rank test by Graphpad. A value of P <0.05 was considered statistically significant. All the statistical analyses were conducted with Graphpad and R 3.3.0 as previously [18].

## Results

### Data information

**Fig. 1:**
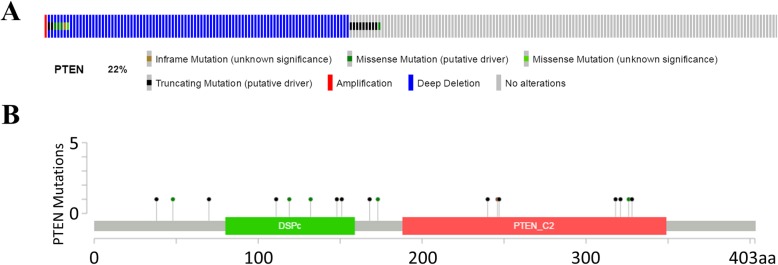
PTEN mutation frequency and types in prostate cancer. A. PTEN mutation frequency in prostate cancer. B. Mutation types of PTEN in prostate cancer. All data were downloaed directly from cBioportal

We downloaded the gene expression data and clinical information of 499 prostate cancer patients from TCGA database (project: TCGA-PRAD). 108 patients (22%) were with PTEN mutation and the rest were PTEN wild type (Fig. 1 a). Mutation types included deep deletion, truncating, and missense mutations spanning over entire gene (Fig. 1 b).

### GSEA

**Fig. 2:**
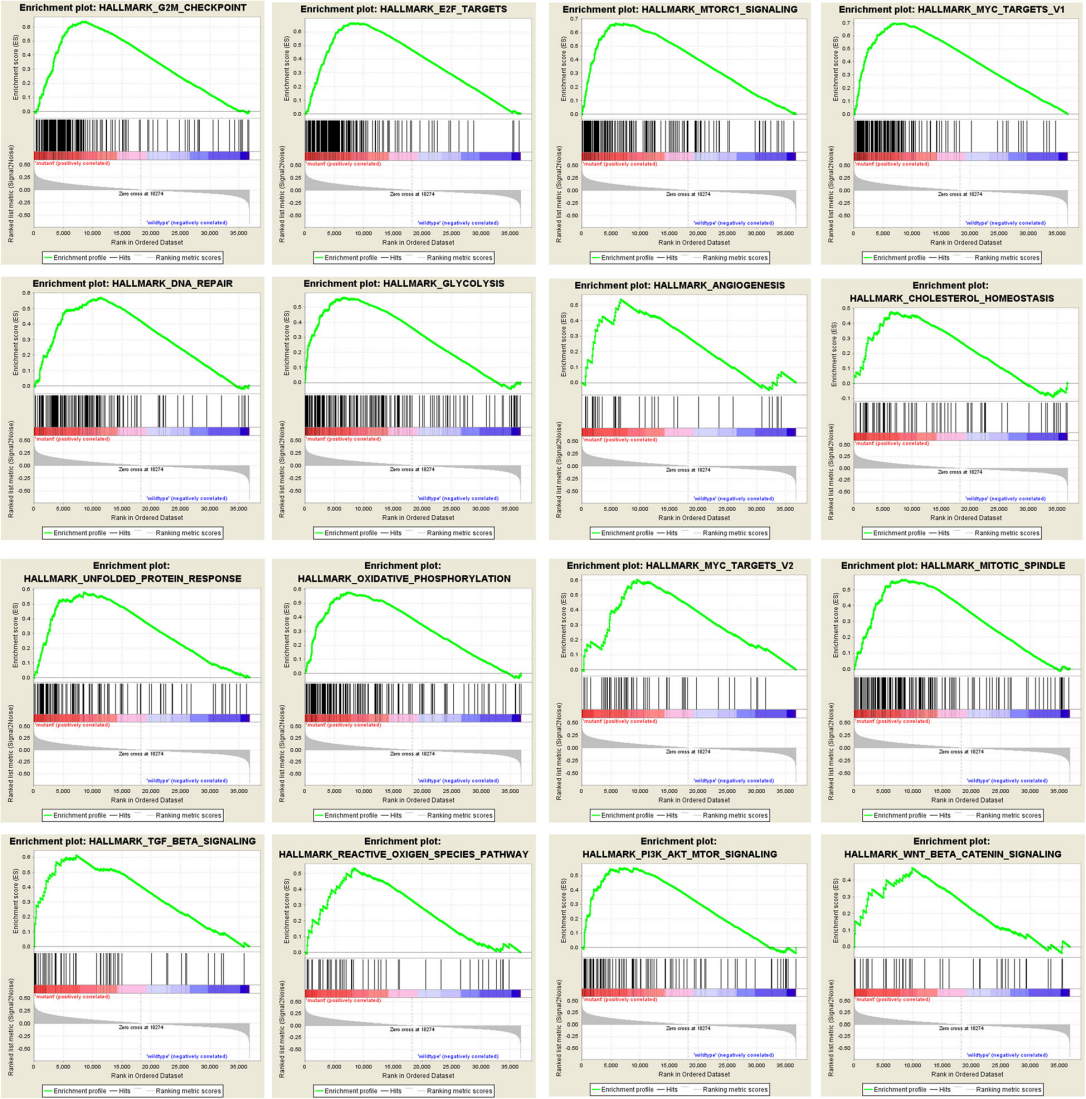
GSEA results showed primary biological functional gene sets enriched in PTEN mutation prostate cancer patients, including G2M check point, E2F targets, mTORC1 signaling pathway, MYC target, DNA repair, glycolysis, angiogenesis, cholesterol homeostasis, unfolded protein response, oxidative phosphorylation, mitotic spindle, TGF-β pathway, reactive oxygen species pathway, PI3K-AKT-mTOR signaling pathway and WNT-β catenin signaling pancreas

To further explore the function of PTEN mutation in disease progression, we analyzed the a variety of biological functional gene sets by the GSEA. As in Fig. 2, results showed that biological processes including G2M check point, DNA repair, glycolysis, angiogenesis, cholesterol homeostasis, unfolded protein response and oxidative phosphorylation were significantly enriched. This suggests that PTEN mutation might infuence disease program by regulating mitosis, DNA repair and metabolism. E2F targets and MYC targets were significantly enriched in GO enrichment analysis. Furthermore, enriched functional pathways included mTORC1 signaling pathway, PI3K-AKT-mTOR signaling pathway, WNT-β- catenin signaling, reactive oxigen species pathway and TGF-β pathway (Fig. 2).

### Clinical affairs of PTEN mutation

**Fig. 3:**
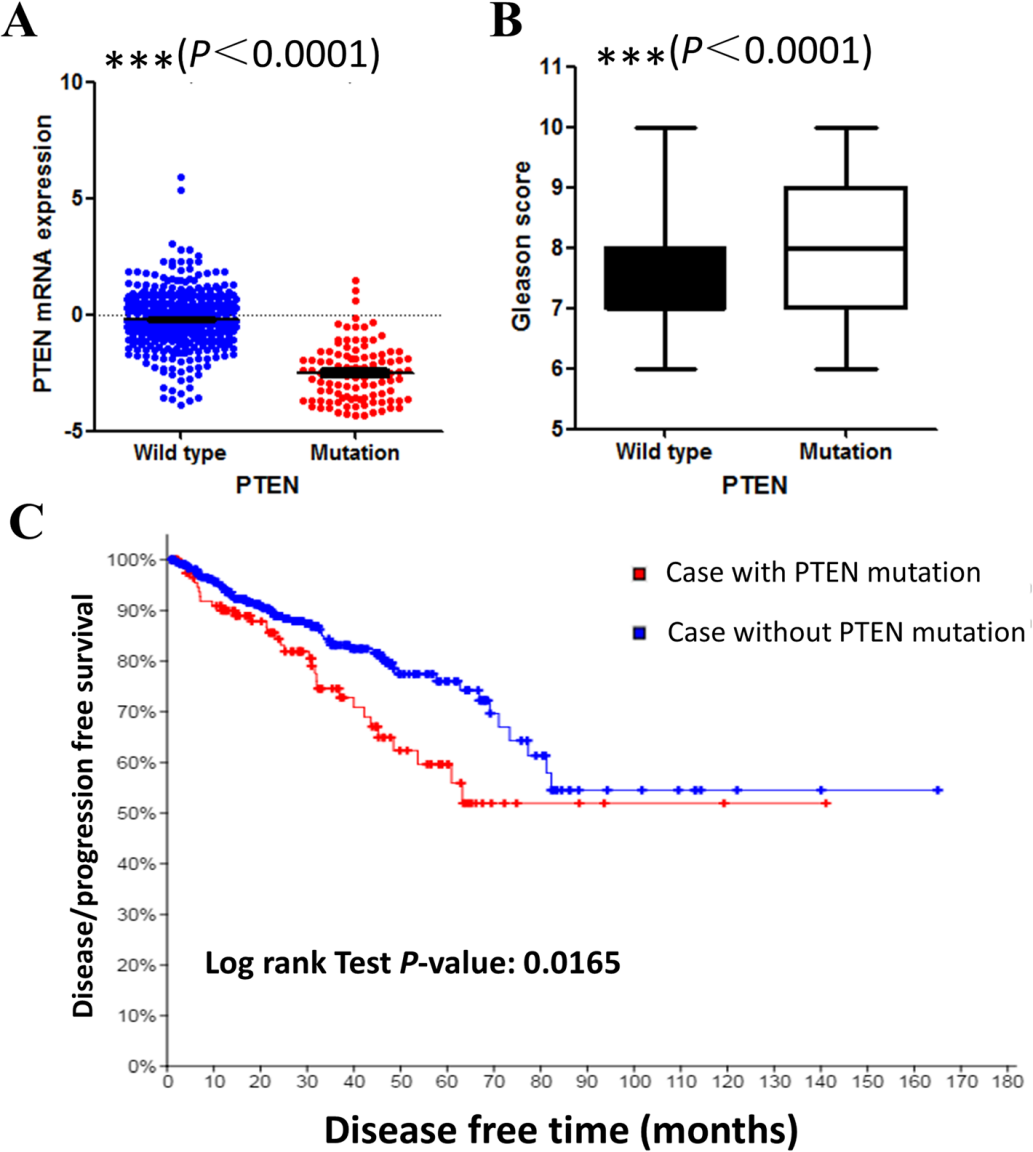
Mutations of PTEN is associated with lower PTEN expression level, higher Gleason score and poorer prostate cancer prognosis. A. Correlation between PTEN mutation and PTEN mRNA expression. B. Correlation between PTEN mutation and Gleason score. C. Survival curves of PCa patients stratified by PTEN mutation

We then focused on the clinical impact of PTEN mutation on prostate cancer. We first explored the PTEN mRNA expression level in both group and found that PTEN was downregulated in tumor tissues with PTEN mutation (Fig. 3 A). Meanwhile, patients with PTEN mutation showed relatively higher Gleason score, which determines the histological grading of prostate cancer (Fig. 3 B). Furthermore, we did Kaplan–Meier analysis to evaluate whether PTEN mutation correlates with and prostate cancer recurrence. Data from cBioportal confirmed that patients with PTEN mutation often have relatively poorer prognosis than those without mutation (Fig. 3 C). All results above indicated the significance of PTEN mutation in prostate cancer clinical impact.

### Identification of DEGs

**Fig. 4:**
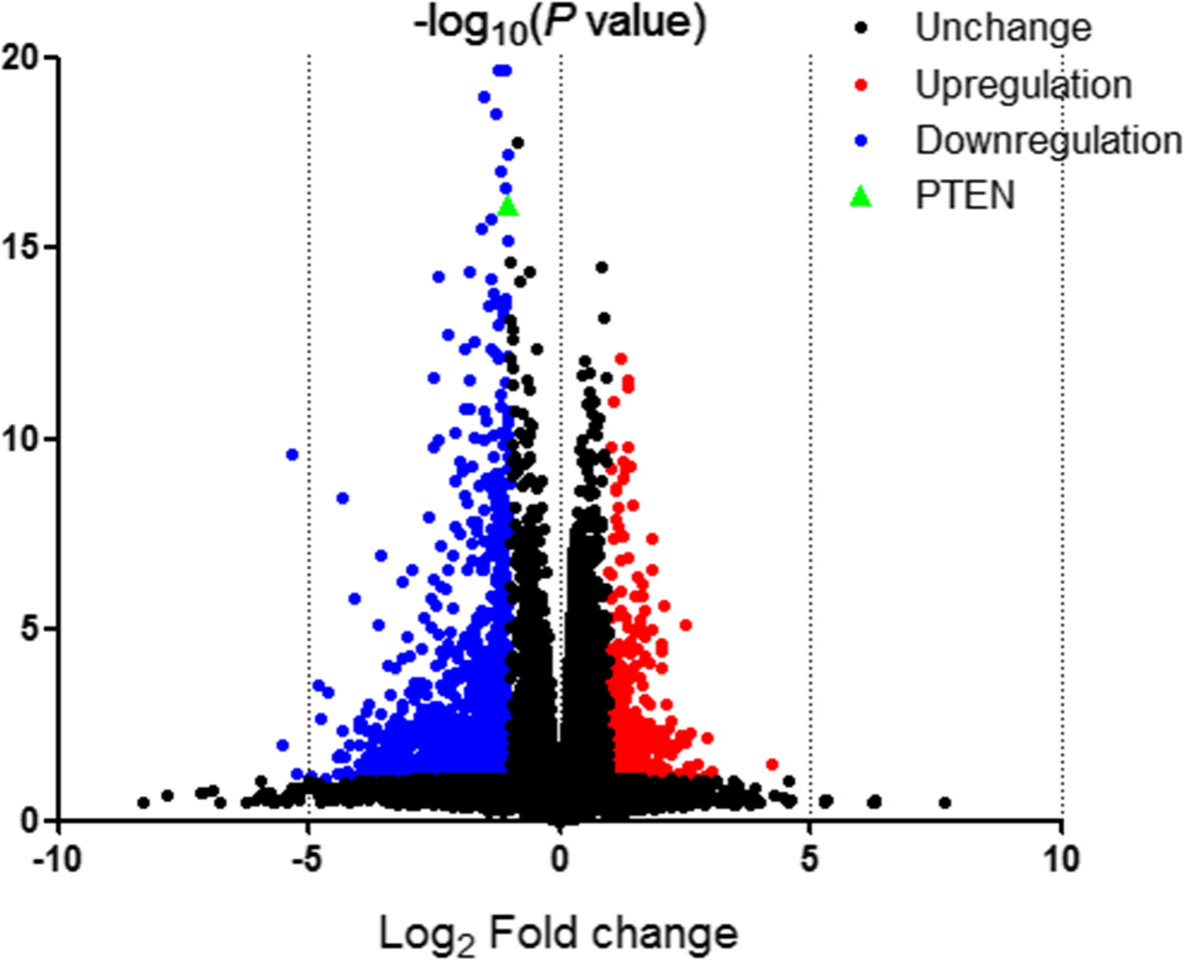
Volcano plot for DEGs. Notes: Red: up-regulated genes; blue: down-regulated genes; black: non-differentially expressed genes; green: PTEN

To further explore the underlying mechanisms by which PTEN mutation influences prostate cancer progression, we identified the DEGs. A total of 1736 genes were identified as DEGs, of which, 344 were upregulated and 1392 were downregulated. Figure 4 shows the volcano plot of the DEGs, while the heat map of top 100 DEGs is shown in Additional file 1: Fig. S1.

### GO and KEGG analysis of DEGs

**Fig. 5:**
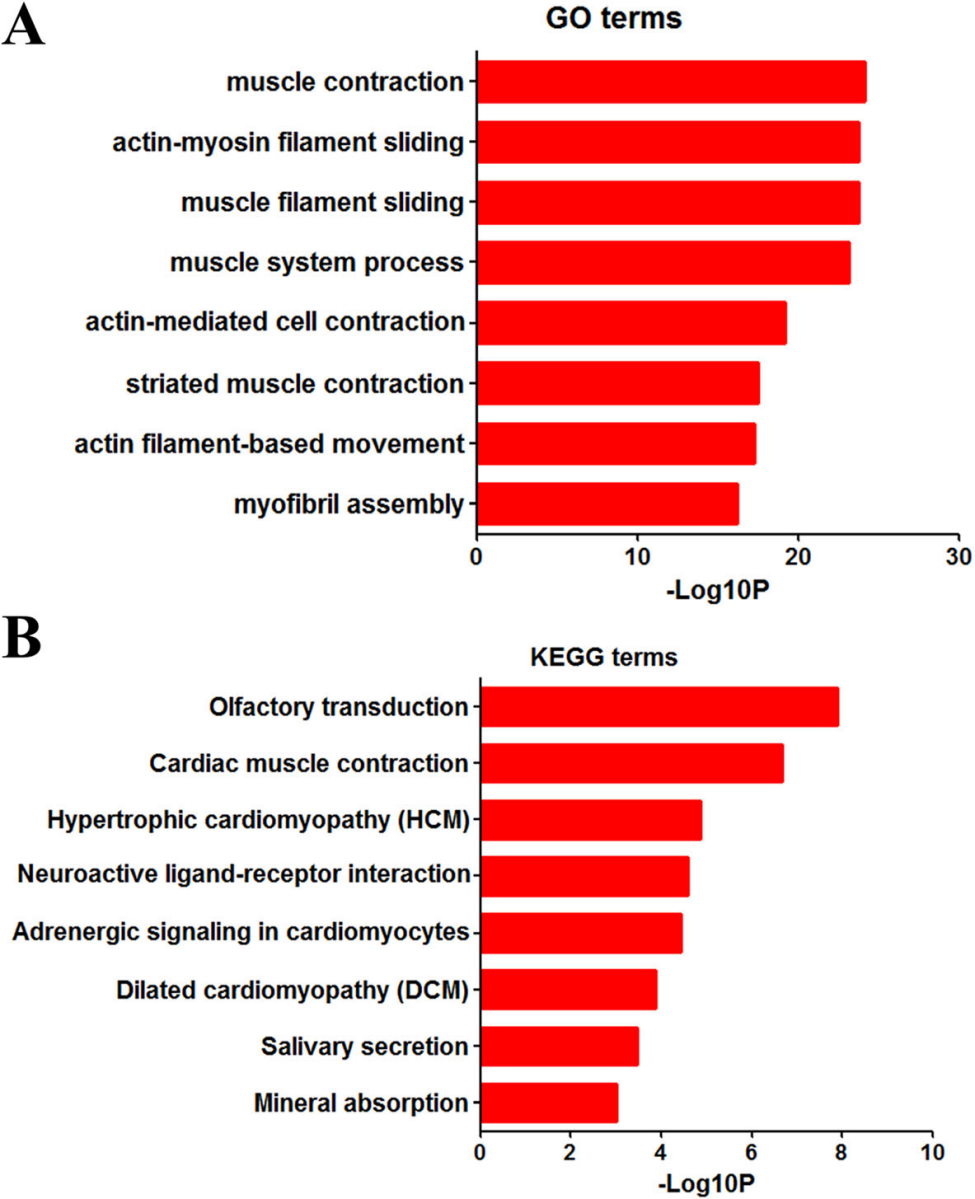
Functional enrichment results of differentially expressed genes. A. Top 8 pathways identified in the GO enrichment analysis in DEGs. B. Top 8 pathways identified in KEGG pathway analysis in DEGs

To further analyze the DEGs at the functional level, we used R package cluster Profiler for our GO/KEGG enrichment analysis to deal with the 1736 DEGs. The GO analysis (Fig. 5 A) suggested significant enrichment in muscle contraction, muscle filament sliding, actin-myosin filament sliding, muscle system process, actin-mediated cell contraction, striated muscle contraction, actin filament-based movement and myofibril assembly.

Furthermore, results of KEGG pathway analysis showed significant enrichment in olfactory transduction, cardiac muscle contraction, hypertrophic cardiomyopathy (HCM), neuroactive ligand-receptor interaction, adrenergic signaling in cardiomyocytes, dilated cardiomyopathy (DCM), salivary secretion and mineral absorption (Fig. 5 B).

### Protein-protein interaction analysis and module screening

**Fig. 6:**
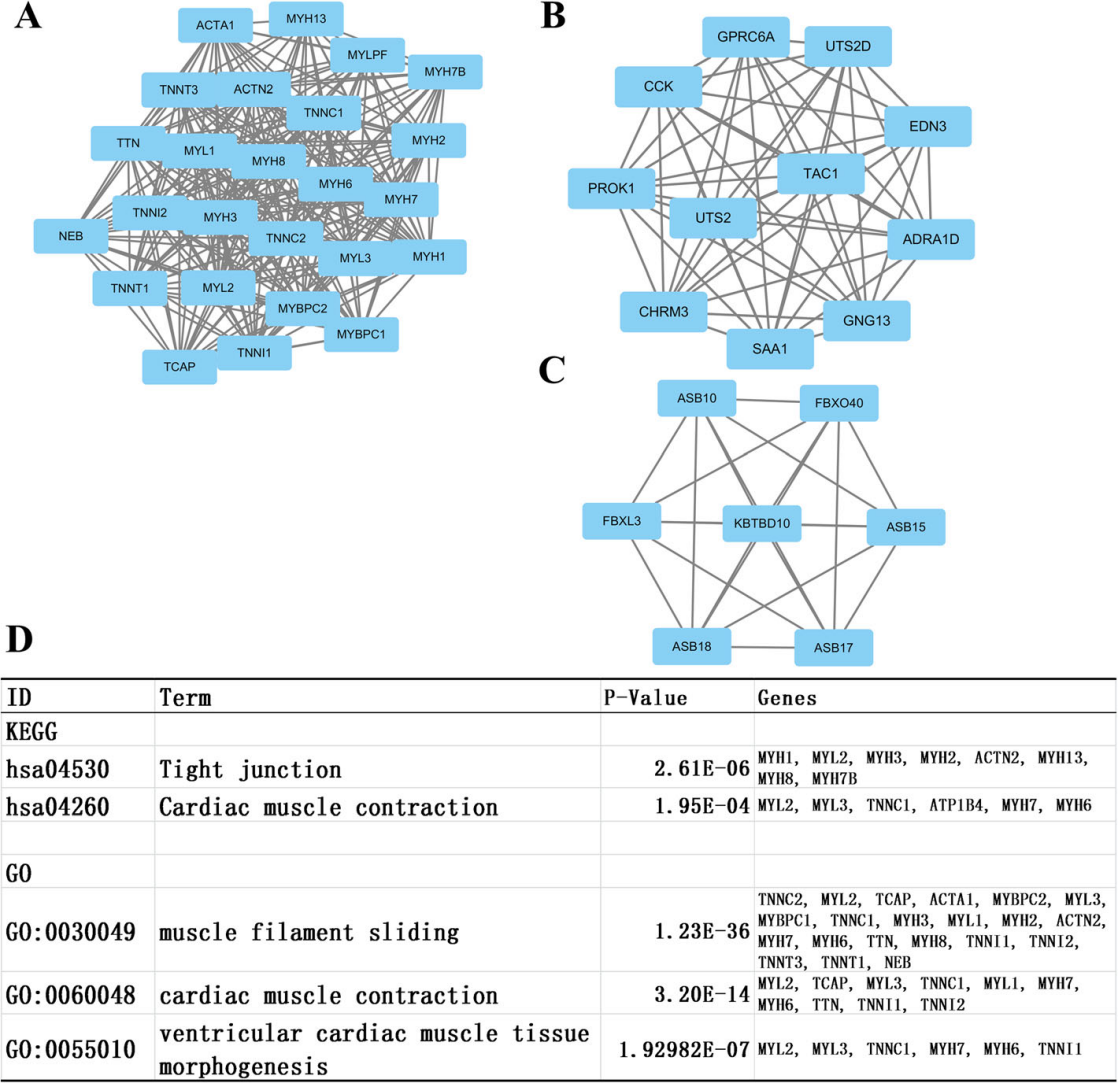
Top three modules from the PPI network and the results of KEGG and GO analyses of module 1. A. Protein interaction network of module 1; B. Protein interaction network of module 2; C. Protein interaction network of module 3; D. GO and KEGG analyses of module 1

We then uploaded all DEGs above to the STRING database to investigate the protein interaction and identify critical hub genes. We found out the top ten genes as hub genes which were ranked by degree. The top 10 hub genes were GNG13, ACTN2, POTEE, ACTA1, MYH6, MYH3, MYH7, MYL1, TNNC1 and TNNC2. GNG13 had the highest degree of nodes with 55. Then, we used the MCODE plugin in Cytoscape to identify the modules of genes in PPI network. Then we did functional enrichment analysis based on the top three significant modules (Additional file 2: Table S1 and Fig. 6, respectively). We selected the top 3 significant modules and did GO/ KEGG analysis. KEGG and GO analyses found enrichment only in module 1. KEGG annotation in module 1 genes was enriched in tight junction and cardiac muscle contraction. GO analysis showed that the genes in module 1 were mainly associated with muscle filament sliding, cardiac muscle contraction and ventricular cardiac muscle tissue morphogenesis.

## Discussion

PTEN, which is a tumor suppressor protein and is very commonly lost across cancer types [19]. The major function of PTEN depends on its phosphatase activity. PTEN mainly inhibits PI3K/AKT pathway activity, while other studies also suggest that PTEN may function through AKT-independent [8]. Mutations in PTEN result in losing its phosphatase activity, which contribute to the onset and progression of tumors and related diseases, including hereditary cancers such as prostate cancer.

Here in our study, we analyzed the gene expression data of prostate cancer obtained from TCGA to uncover the critical pathways and top hub genes associated with PTEN mutation. We found 22% patients with PTEN mutation among all cases. The mRNA expression and clinical affair analyses showed lower expression level of PTEN, higher Gleason score and poorer prognosis in patients with PTEN mutation, which indicated the significance of PTEN mutation in prostate cancer: PTEN mutation correlated with advanced disease and worse outcome. PTEN deletion, transcriptional and epigenetic modifications of PTEN are known mechanisms that could cause deregulation of PTEN [20, 21]. Lower PTEN expression level often correlates to disease progression in various cancer types [22–24], which makes it one of the important potential mechanisms how PTEN mutation exert the role in disease progression. In the following research, the relationship between PTEN mutation and more details of clinical affairs of prostate cancer requires larger sample data for more accurate results.

Now that PTEN mutation contributes to prostate cancer progression, the mechanisms underlying other than PTEN downregulation are critical. GSEA analysis suggests that PTEN mutation were mainly associated with the cell metabolism, proliferation and cancer related pathways. In the cellular processes above, metabolic processes, including glycolysis and lipid metabolism, always play critical roles in cancer progression. Previous studies have shown that the formation of cancer cells requires adaptations across various metabolic processes to satisfy the energy required for their increased rate of proliferation. Dysregulation of lipid metabolism, including upregulation of several lipogenic enzymes, has been a hallmark of prostate cancer, and metabolic target has been shown to be a potential treatment target in prostate cancer [25, 26]. Our results indicated that drugs targeting lipid metabolic pathways could contribute to the development of new therapeutic modalities in PTEN mutation prostate cancer patients. As for DNA repair, previous researches have suggested that mCRPC has genomic aberrations that implicated in DNA repair [27, 28]. Poly (adenosine diphosphate [ADP]–ribose) polymerase (PARP) is a critical factor that functions in the biologic progression of DNA repair, and PARP inhibition has been shown to exert anti-tumor activity in sporadic mCRPC cases by treating DNA repair defects in tumor cells [29]. In addition, antiandrogen therapy, the main strategy for the treatment of prostate cancer, could significantly lead to increased DNA damage and reduced colony formation survival. Our findings suggested that mechanisms implicated with DNA repair might contribute to disease progression, thus providing new insights into the development mechanisms of prostate cancer.

Today, the detection of mutations in important tumor genes and tumor suppressor genes has been applied clinically. These test results can help clinicians select more effective individualized treatment strategies. Our results indicate that G2M check point is one of the biological processes significantly enriched in patients with PTEN mutation. As we all know, first-generation taxane chemotherapies, Docetaxel (Taxotere), which is the first-line drug in prostate cancer chemotherapy, effectively target the cytoskeleton by stabilizing the interaction of β-tubulin subunits of microtubules preventing depolymerization, inducing G2M arrest and apoptosis [30]. This suggests that patients with PTEN mutation may be more sensitive to Docetaxel. Moreover, our results above have shown that PTEN mutation correlated with advanced disease and worse outcome. Combining these results, we provide the evidence that early intervention is required for patients with PTEN mutation to obtain a longer survival period, and that chemotherapies using Docetaxel might be more effective in such patients, which provides a potential proof for individualized treatment.

We then identified the DEGs in both groups and carried out the functional analysis. 1736 genes in all were identified as DEGs with different PTEN mutation status. Enrichment analysis indicated that DEGs in PTEN mutation patients contributed to cell movement, tight junction and cell growth, which implies that cancer cells of patients with PTEN mutation might have greater mobility and migration.

In protein-protein interaction analysis, we identified the top 10 genes as the hub genes. GNG13, which encodes G Protein Subunit Gamma 13, functions as signal transducer for the 7-transmembrane-helix G protein-coupled receptors [31]. GO annotations related to this gene include obsolete signal transducer activity and G-protein beta-subunit binding. In previous studies, the dysregulation of GNG13 was only found to be involved in the disease program of breast cancer [32]. Perhaps in the future, in-depth research will unearth the important role of this hub gene in prostate cancer. Other hub genes include ACTN2, POTEE, ACTA1, MYH6, MYH3, MYH7, MYL1, TNNC1 and TNNC2. One or several of the genes above might become new targets for prostate cancer research in the future.

Our study contained one limitation. In our study, we conducted a preliminary research on the effects of PTEN mutation in the progression and prognosis of prostate cancer. The mechanism and validation of PTEN mutations in prostate cancer still need further research in clinical and molecular biology experiments.

## Conclusions

This study found out the main pathways and hub genes associated with PTEN mutation in prostate cancer. PTEN mutation is associated with advanced disease progression and the poorer prognosis of prostate cancer. Our results may facilitate the identification of PTEN mutation as a subgoup to improve prostate cancer prognosis prediction and develop therapeutic strategies.

## Supplementary information

**Additional file 1: Fig. S1.** Heat map of the top 100 differentially expressed genes. Red: up-regulation; purple: down-regulation.

**Additional file 2.** Hub genes list in protein-protein interaction analysis.

## Data Availability

Not applicable.

## Abbreviations

DAVID: Database for annotation, visualization and integrated discovery
DEG: Differentially expressed gene;
GEO: Gene Expression Omnibus;
GO: Gene ontology
GSEA: Gene set enrichment analysis;
KEGG: Kyoto Encyclopedia of Genes and Genomes
MCODE: Molecular Complex Detection
PPI: Protein–protein interaction;
PTEN: Phosphatase and Tensin Homolog;
RNA-Seq: RNA sequencing;
STRING: Search Tool for the Retrieval of Interacting Genes/Proteins
TCGA: The Cancer Genome Atlas
