# Supplementary figures and images for: Identification of key pathways and genes in PTEN mutation prostate cancer by bioinformatics analysis
Source: BMC Med Genet. 2019 Dec 2;20:191. doi: 10.1186/s12881-019-0923-7 (PMC6889628; doi:10.1186/s12881-019-0923-7)

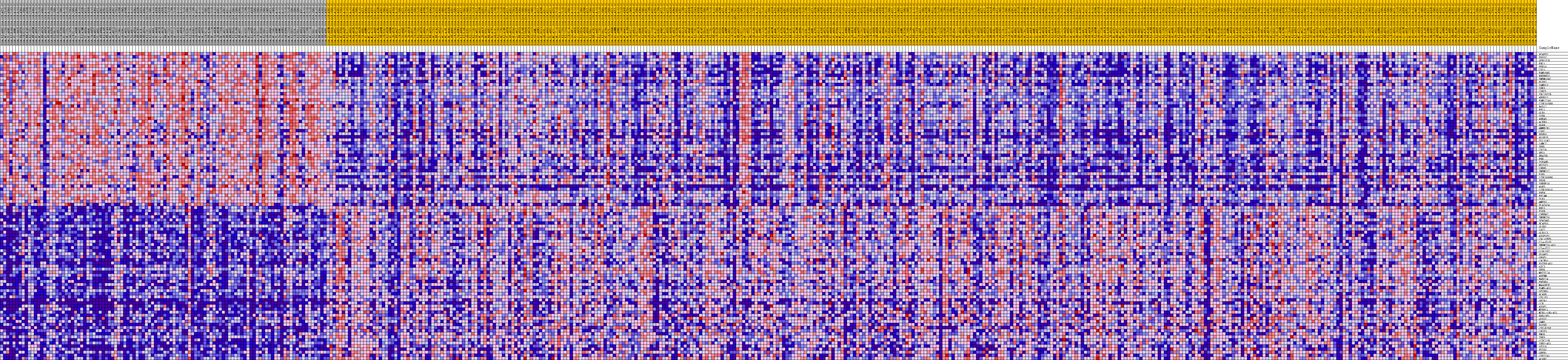

Supplement: Supplementary file 1 — Additional file 1: Fig. S1. Heat map of the top 100 differentially expressed genes. Red: up-regulation; purple: down-regulation. [file 12881_2019_923_MOESM1_ESM.jpg]
